# The effects of a Cognitive Stimulation Therapy [CST] programme for people with dementia on family caregivers’ health

**DOI:** 10.1186/1471-2318-14-31

**Published:** 2014-03-14

**Authors:** Elisa Aguirre, Zoe Hoare, Aimee Spector, Robert T Woods, Martin Orrell

**Affiliations:** 1 Unit of Mental Health Sciences, University College London, Charles Bell House, 67-73 Riding House Street, London W1W 7EJ, England; 2 Research and Development Department, North East London Foundation Trust, Goodmayes Hospital, Barley Lane, Ilford, Essex, England; 3 North Wales Organisation for Randomised Trials in Health [NWORTH], Institute of Medical & Social Care Research, Bangor, Wales; 4 Research Department of Clinical, Educational and Health Psychology, University College London, 1-19 Torrington Place, London WC1E 7HB, England; 5 DSDC Wales, Bangor University, 45 College Road, Bangor, Gwynedd LL57 2AS, Wales

**Keywords:** Dementia, Alzheimer’s disease, Family caregivers, Cognitive stimulation, Quality of life

## Abstract

**Background:**

There is growing evidence that Cognitive Simulation Therapy (CST) benefits cognition and quality of life of people with dementia, but little is known about the indirect effects of this intervention on family caregivers. This study sought to investigate the effect of CST on family caregivers general health status of people with dementia living in the community attending the CST intervention.

**Method:**

Eighty-five family caregivers of people with dementia took part in the study. All the people with dementia received the standard twice weekly seven weeks of the CST intervention plus either 24 weeks of a maintenance CST (MCST) intervention or 24 weeks of treatment as usual. Family caregivers were assessed before and after their relatives the CST programme, and after 3 and 6 months of the MCST programme. A pre and post CST groups comparison was undertaken to evaluate the open trial first phase and an ANCOVA model used to analyse the maintenance phase with its controlled comparison.

**Results:**

We found no evidence for a benefit on the family caregiver outcome measures of the intervention before and after CST groups by using a t-test analysis or any significant differences between intervention and control groups for any of the variables considered at any time point (3 and 6 month follow up).

**Conclusion:**

CST seems to have a relatively specific benefit fpr people with dementia that may not carry over to family carers. Future studies need to further explore and compare the effects that CST might bring to family caregivers of people with dementia attending the intervention.

**Trial registration:**

Current Controlled Trials ISRCTN26286067

## Background

It has been estimated that currently 35.6 million people live with dementia and this number will increase in 2050 by 115.4 million [1]. The majority of people with dementia are cared for at home and looked after by a member of the family or close friend [2] and therefore, as the number of people with dementia increases so will the number of family caregivers. Being a carer for people with dementia has been associated with frequent use of medication and increased visits to health professionals [3,4], as well as with poor clinical outcomes such as depression, illness and decreased quality of life [5]. Dementia caregiving has also been linked with poor outcomes for people with dementia such as poor quality of life and increased risk of nursing home placement [6,7].

Systematic reviews have found cognitive stimulation to be a psychosocial intervention for people with dementia [1,8-10], which has robust evidence. Cognitive stimulation programmes target cognition but have a social element usually in a group or with the family caregiver, and the included cognitive activities do not primarily consist of practice on specific cognitive modalities [11].

Evidence in relation to its effectiveness on family caregiver outcome measures is less conclusive. The Cochrane review found only three studies reporting on family caregivers outcomes and in these studies [12-14], no differences were noted and the effect sizes for anxiety, depression and caregiver burden were close to zero, indicating no likely differences in caregiver outcomes [10]. In one study [12], the family caregivers were trained to deliver the intervention, without showing any adverse effect on them [10].

Generally the effectiveness of interventions has been judged in relation to how far the specific outcomes relate to the specific person targeted (person with dementia or family caregiver). However, there is little evidence as to whether interventions targeted at people with dementia might benefit carers. So far the dominant conceptual model for caregiving is the stress-coping model [15] that assumes that the onset and progression of chronic illness and physical disability are stressful for both the person with dementia and the family caregiver [15]. Therefore, under this framework, it is expected that CST by increasing the person with dementia’s cognitive abilities and well being, may lead to an improvement in family caregivers mood and wellbeing.

The aim of this study is to examine the impact on the family caregivers of the CST programme for people with dementia.

## Methods

### Participants

Participants were family caregivers of people with dementia living in the community who were taking part in a large randomised control trial of Maintenance CST (MCST) for dementia [16,17]. Ethical approval was obtained through the Multi-centre Research Ethics Committee (ref no. 08/H0702/68). The clinical trial is registered – ISRCTN26286067. Family caregivers were recruited from January 2009 till September 2010 in community settings in London, Essex and Bedfordshire. Family caregivers were referred by the manager from the day centre or community mental health team where the CST trial groups were taking place. Family caregivers were contacted and invited for a meeting where the purpose of the trial was explained and if consented were recruited with their relatives for the study.

### Design

**Figure 1:**
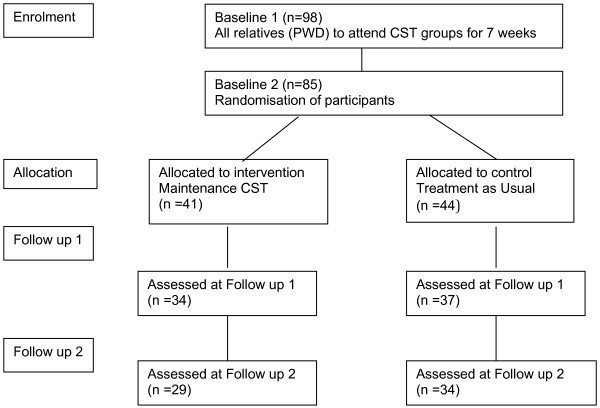
Trial flow chart.

People with dementia received standard CST which is a seven-week 14-session group psychosocial treatment for dementia that is effective in improving cognition and quality of life [18]. They were then randomised into either [a] the maintenance CST programme once weekly for 24 weeks or [b] treatment as usual [17] (Figure 1). Assessments of the family caregivers were conducted in the week prior to and following their relatives participation in the standard CST intervention, and at three and six month follow up after randomisation into the maintenance programme group or treatment as usual.

### Intervention

The intervention included the standard CST programme, 14-sessions run twice weekly [19,20] followed by the Maintenance CST programme [21,22] or treatment as usual. The intervention was based on the theory of cognitive stimulation as applied to the original CST programme [19] guided by the MRC framework [23] for complex interventions. The complete CST and Mainteance CST programme, incorporated the use of an “RO board”, displaying both personal and orientation information, including the group name (as chosen by participants). The guiding principles of the intervention involved using new ideas, thoughts and associations; using orientation sensitively and implicitly; a focus on opinions rather than facts; using reminiscence as an aid to the here-and-now; providing triggers to aid recall; creation of continuity and consistency between sessions; focus on implicit (rather than explicit) learning; stimulating language; stimulating executive functioning and being person-centred (treating people as unique individuals with their own personality and preferences). Each group had two facilitators, one from the research team and a co-facilitator who was a member of staff from the recruited centre (e.g. residential care home). The use of two facilitators for each group enabled effective de-briefing and reflection to occur at the end of each session.

### Assessment measures

#### *EQ-5D*

EQ-5D [24] is a standardized instrument for use as a measure of health outcome. Applicable to a wide range of health conditions and treatments, it provides a simple descriptive profile and a single index value for health status. EQ-5D was originally designed to complement other instruments but is now increasingly used as a 'stand alone' measure. EQ-5D is designed for self-completion by respondents, and is easy, taking only a few minutes to complete. Instructions for family caregivers to follow were included in the questionnaire. The scale includes a 3- level (1 = no problem, 2 = moderate problem, 3 = severe problem), 5-dimensional format and the Visual Analogue Scale [VAS] with a maximum score of 100 indicating the best health status.

#### SF-12

The Short Form-12 UK [25] Health Survey measures generic health concepts relevant across age, disease, and treatment groups. It provides a comprehensive, psychometrically sound, and efficient way to measure health from the patient's point of view by scoring standardized responses to standard questions. The SF-12 includes 8 concepts commonly represented in health surveys: physical functioning, role functioning physical, bodily pain, general health, vitality, social functioning, role functioning emotional, and mental health.

### Analysis

For the before and after CST data the analysis followed a paired sample t-test analysis. For the maintenance CST or treatment as usual phase, an ANCOVA model was fitted for each of the follow up time points. The ANCOVA model incorporated centre as a random factor and baseline 2 score (post CST) as a covariate.

## Results

Tables 1 and 2 presents baseline data before and after the CST groups on sociodemographic and service use variables. Generally participants were female (n = 84; 86%), above retirement age, married to the person with dementia (n = 44; 45%) or son/daughters (n = 47; 48%). Almost all were providing daily assistance to people with a diagnosis of Alzheimer’s type of dementia (n = 45; 46%). The mean age of the people with dementia was at 81 [range 52–97] years. Overall retention was good. We followed up 71 (83.5%) participants at 3 months and 63 (74.1%) at six months.

**Table 1:** Sociodemographic characteristics at baseline 1 (n = 98)

**Characteristics**	
Relationship to PWD at BL1 –	
Spouse	44 (45%)
Child/niece/nephew)	47 (48%)
Brother/sister	2 (2%)
Friend	3 (3%)
Other (Grand-daughter, Warden)	2 (2%)
Carer living situation	55% Lives in
Type of dementia	
Alzheimer’s type	45 (46%)
Vascular type	16 (16%)
Other	15 (15%)
Not known	20 (20%)
Missing	2 (2%)
Carer employment status	26 retired
	28 employed
	5 unemployed
	3 housewife/husband
	36 missing

**Table 2:** Sociodemographic characteristics at baseline 2 (n = 85) divided by trial arm

		**MCST**	**TAU**	**Total**
Relationship to PwD at BL1	Spouse	19	23	42
	Child (inc. niece/nephew)	21	17	38
	Brother/Sister	0	2	2
	Friend	0	1	1
	Other (Grand-daughter, Warden)	1	1	2
	Lives with	24	26	50
	Doesn’t live with	17	18	35
Type of dementia	Alzheimer’s type	12	25	37
	Vascular type	8	4	12
	Other	10	5	15
	Not known	9	10	19
	Missing	1	1	2
Carer Status	Retired	7	19	26
	Employed	12	10	22
	Unemployed	4	1	5
	Housewife/Househusband	0	1	1
	Missing	13	15	28

Table 3 shows the analysis of the first phase of the study. There was no evidence for a benefit of intervention immediately after the standard CST group programme on the family caregiver outcome measures. Table 4 shows the analyses of the ANCOVA for the Maintenance stage of the programme. Again, we found no evidence of any significant differences between groups for any of the variables considered at any time point. [3 and 6 month follow up].

**Table 3:** Family caregiver outcomes before and after CST (Baseline 1 to Baseline 2)

**Measure**	**n for CC**	**Before CST mean (SE)**	**After CST mean (SE)**	**Difference**	**t value**	**p value**	**Effect size**
SF-12b PCS	85	38.14 (6.39)	38.84 (6.31)	0.70 (6.01)	1.071	.29	0.12
SF-12 MCS	85	42.25 (5.25)	42.33 (5.13)	0.08 (5.62)	0.134	.89	0.01
EQ-5Db Utility	82	0.82 (.21)	0.84 (0.21)	0.02 (.205)	0.78	.44	0.08
EQ-5Db VAS	82	71.73 (16.85)	74.61 (18.26)	2.87 (16.08)	1.62	.11	0.18

**Table 4:** Effects of Maintenance CST on adjusted complete case (CC) outcomes at primary and secondary end points

	**FU1- 3 month follow up**					**FU2- 6 month follow up**				
	**Treatment**		**Control**	**Difference**	**p value**	**Treatment**		**Control**	**Difference**	**p value**
Adjusted outcomes	n	Mean (SE)	Mean (SE)	Mean (SE)		n	Mean (SE)	Mean (SE)	Mean (SE)	
SF-12 PCS	71	39.07 (0.93)	39.26 (0.97)	0.19 (1.34)	.89	63	37.89 (0.94)	38.44 (1.02)	0.55 (1.39)	.70
SF-12 MCS	71	42.43 (0.76)	43.04 (0.80)	0.61 (1.11)	.58	63	41.88 (0.85)	42.91 (0.92)	1.03 (1.26)	.42
EQ-5D Utility	70	0.83 (0.02)	0.81 (0.02)	−0.02(0.03)	.52	63	0.79 (0.02)	0.84 (0.02)	0.05 (0.03)	.12
EQ-5D VAS	70	75.86 (2.05)	74.04 (2.17)	−1.82 (2.99)	.57	63	74.41 (2.33)	72.28 (2.52)	−2.14 (3.43)	.54

## Discussion

This study evaluated the potential indirect effects of CST on the family caregivers general health status and quality of life. The standard twice weekly for seven weeks CST intervention showed benefits to cognition and quality of life for people with dementia [18] and the maintenance programme showed to benefit quality of life of people with dementia at 6 months [19] and cognition for people on cholinesterase inhibitors [19]. However, we found no evidence for a benefit on the family caregiver outcome measures.

The external validity of this trial is high though two aspects limit its generalisability, lack of ethnic mix and wide geographical spread. Participants were almost exclusively white British and therefore we cannot draw conclusions on the effectiveness of this intervention for other ethnical backgrounds or cultures. In addition, it may be that an effect would be found with a larger numbers of family carers.

Research so far suggests two basic types of interventions to help and support people caring for a relative or a friend with dementia. These range from those [a] providing direct practical assistance with care by the provision of respite care, to [b] interventions directly delivered to family caregivers by offering psycho educational or psychosocial support. Non-cognitive features of dementia are most likely to be associated with psychological problems in caregivers [26]. The stress/health model as the dominant theoretical model guiding the design of caregiver interventions, assumes that the severity of dementia is one of the primary stressors on the caregiver [27]. Thus, our study hypothesized that if CST can improve cognitive abilities and increase their quality of life, this effect could potentially have a beneficial effect on family caregivers. As the perceived health of the family caregivers is not only related to cognitive impairment of the person with dementia, but also to the behavioural and psychological symptoms in dementia [26], further studies should include these variables in their analysis when evaluating further the indirect effect of this intervention.

Different types of interventions for people with dementia may be more or less effective depending upon the nature of the relationship between the caregiver and the care recipient. It might be that a cognitive based intervention delivered by a family caregiver such as individualized CST [28] might be of greater success in positively influencing both partners. In a pilot study, Moniz-Cook et al. [29] found that a home-based memory management programme involving the family carer led to improvements in memory in the person with dementia, improvements in carer wellbeing, and a reduction in care home admissions at 18 months follow-up. Similarly benefits in cognition in people with dementia and carer wellbeing have been reported in studies by Quayhagen et al. [30].

## Conclusions

Group CST interventions benefit people with dementia but there is little evidence to suggest that they may be beneficial for family caregivers. Conclusions from this study are only preliminary and therefore further studies need to improve the methodological qualities of this type of study, for instance, increasing sample size and measuring a wider range of family caregivers outcomes (eg. burden, knowledge, anxiety, depression, quality of life). Further studies could also explore the impact of individual CST on different family caregiver outcomes.

## Competing interest

AS runs the CST training course on a commercial basis. EA, AS, BW and MO have co-authored the CST and Maintenance CST manual, the royalties from which are received by the Dementia Services Development Centre Wales.

## Authors’ contributions

All authors participated and contributed to design and conduct of the SHIELD-Maintenance CST trial and commented on drafts, read and approved the final manuscript. EA lead the writing of this paper and wrote the first draft of the presented manuscript, implemented the design and overall organisation of the trial; managed the development of the programme; recruited all centres that took part in the trial; recruited and assessed participants and ran some of the intervention groups. ZH was trial statistician and led data management and analysis of the trial. AS was a co-applicant and provided clinical supervision for the researchers running the intervention and contributed to the theory behind the trial. RTW was co-applicant and principal investigator for clinical psychology. MO was principal applicant and chief investigator; he led the design and execution the trial.

## Pre-publication history

The pre-publication history for this paper can be accessed here:

http://www.biomedcentral.com/1471-2318/14/31/prepub
